# Supplementary material for: Memory and Perception-based Facial Image Reconstruction
Source: Sci Rep. 2017 Jul 26;7:6499. doi: 10.1038/s41598-017-06585-2 (PMC5529548; doi:10.1038/s41598-017-06585-2)
Supplement: Supplementary file 1 — Supplementary Information [file 41598_2017_6585_MOESM1_ESM.pdf]

Supplementary Information

Memory and Perception-based Facial Image Reconstruction

Chi-Hsun Chang[1]*, Dan Nemrodov[1], Andy C. H. Lee[1-2‡], and Adrian Nestor[1‡]

1. Department of Psychology at Scarborough, University of Toronto, Toronto, Ontario, Canada
2. Rotman Research Institute, Baycrest Centre, Toronto, Ontario, Canada

*Correspondence concerning this article should be addressed to Chi-Hsun Chang Department of Psychology at Scarborough, University of Toronto, Toronto, Ontario, M1C 1A4, Canada. Email: ch.chang@mail.utoronto.ca

‡ Reflects equal contribution

Stimuli. Sixty unfamiliar face images were selected from multiple face databases while thirty images of media celebrities (i.e., actors and politicians) were selected from freely-available online public sources. All images displayed front views of adult Caucasian males with a neutral expression and no hair or facial accessories. All images were cropped to reveal only internal features of the face, spatially normalised, based on the position of the eyes and the nose, and colour-normalised by equating their mean and root mean square (RMS) contrast values separately for each colour channel in CIEL*a*b* colour space.

Screening. Imagery abilities (Vividness of Visual Imagery Questionnaire - VVIQ2[1]), face recognition abilities (Cambridge Face Memory Test – CFMT[2]) and familiarity with famous faces on a 1-7 rating scale (1: not familiar; 7: very familiar) were assessed across multiple healthy adults who volunteered for the study in exchange for payment and/or course credit.

To ensure that participants had good visual imagery and face memory, eligibility for Experiment 1 was determined as follows: (i) VVIQ2 score (range 1-5) > 3; (ii) overall CFMT accuracy > 80%; (iii) old-new recognition accuracy with learned faces > 95% (chance performance: 50%); and (iv) familiarity ratings for at least 3 famous faces > 5, along with their accurate naming. Out of 13 volunteers for the study, three participants satisfied these criteria and proceeded to further testing in Experiment 1. The relatively low passing rate was due to the implementation of strict screening criteria for the purpose of maximising the probability of successful reconstruction and the informational content of reconstruction outcomes.

Further, eligibility for Experiment 2 was determined based on two criteria: (i) CFMT accuracy within the norms of typical abilities[2] and (ii) high familiarity with at least one famous

face triplet from Experiment 1 (i.e., average ratings above 3 on a 7-point scale along with accurate naming). Out of 74 volunteers, 30 satisfied the criteria above and proceeded to further testing. With regard to the second criterion, familiarity estimates (mean ± 1SD) with famous faces within each of the three resulting participant groups, corresponding to NC/CB/SA's target faces, were: 6.4 (± 0.96), 6.4(± 0.49), 6.7(± 0.40).

Novel face learning. Participants were presented with three target faces, side by side, for as long as they deemed necessary and asked to commit them to memory in as much visual detail as possible – this procedure was repeated multiple times throughout the experiment, on each day of testing, so as to refresh and to consolidate their memory of these faces. To the same end, participants also performed an old-new recognition task and a visual identification task with the same target faces as follows.

Participants were presented with either the three learned faces (19 trials each) or the remaining 57 unfamiliar faces (1 trial each) and were asked to press one of two keys accordingly – each stimulus face was presented for 400 ms and subtended 2.4° x 4° at the centre of the screen from a distance of 90 cm. As expected, all participants performed the task well above chance (mean accuracy NC: 99%, CB: 96% and SA: 99%).

Further, participants were tested in an individuation task in which they pressed a key associated with each learned face in response to degraded face displays (40 trials per learned face). The goal of this task was to ensure that participants commit target faces to memory with extensive pictorial detail allowing recognition even in challenging viewing conditions. Specifically, each face image was combined with white noise in various proportions ranging between 50-100% – this proportion was determined adaptively using QUEST[3] so as to maintain

a 75% level of recognition accuracy. In these conditions, all participants were able to perform the task in the presence of a considerable amount of noise (the proportion converged to 82%, 77% and 85% for NC, CB and SA, respectively).

Face space and facial feature derivation. To estimate each participant's face space and to derive significant visual features underlying the organisation of this space, the following procedure was performed.

First, the similarity ratings among the 57 unfamiliar faces obtained from the perception-based similarity rating task were organised into a confusability matrix, where each row and column represent a single face and cells estimate the similarities between pairs of faces (Fig. 1b). Specifically, the similarity score of each face pair, ranging from 1 to 7, was converted into confusability index by subtracting it from 7 and dividing the result by 6 such that all values in the matrix were scaled between 0 and 1.

Second, a face space construct was computed by applying multidimensional scaling (MDS) to the confusability matrix (Fig. 1c). Specifically, metric MDS was deployed to generate a multidimensional space exhibiting Euclidean properties traditionally associated with the topography of psychological face space[4]. This analysis interprets dissimilarities from the confusability matrix as Euclidean distances between pairs of faces and accounts for them as distances in a space with the smallest possible number of dimensions. For the purpose of our analysis we selected a maximum number of 20 dimensions given that: (i) they accounted for a considerable proportion of the variance in our data (>90% for every participant) and (ii) a similar number of dimensions was found sufficient for reconstruction purposes in previous work[5].

Third, for each dimension of the face space, coefficients were z-scored. Normalised coefficients were then divided into positive and negative values and two average face templates were constructed by combining all faces weighted by their coefficients on the positive and negative side of the origin separately. A classification image (CIM) for each dimension was created then by computing the differences between the corresponding positive and negative averaged face templates (raw CIM's in Fig. 1d). We note that this procedure is analogous to reverse correlation techniques used in the study of vision[6–8] in that it aims to derive visual features by averaging stimuli proportionally to the experimental responses that those stimuli elicit; however, unlike typical reverse correlation methods, our approach uses noise-free visual stimuli and it weights stimuli proportionally to MDS coefficients extracted from experimental responses. To be clear, each CIM is, thus, a weighted average of face images attempting to account for the organisation of these images on each dimension of face space – the location of a face on a given dimension is explained by the extent to which that face instantiates the CIM corresponding to that dimension. Naturally, since different dimensions organise faces differently (i.e., by exploiting the ability of MDS to extract uncorrelated dimensions), we expect that CIM's for different dimensions capture different sources of information.

Last, we note that not all dimensions can be expected to encode relevant pictorial information. For instance, some dimensions may encode higher-level semantic information or visual information that cannot be captured well through our method such as higher-frequency spatial information (e.g., skin texture). Accordingly, CIM's were assessed pixel by pixel via a permutation test in order to determine which CIM's contain significant visual information. Accordingly, for each dimension, all coefficients of the 57 unfamiliar faces were randomly permuted 10,000 times and a CIM was generated for every permutation. The value of each pixel

in the original CIM was then compared relative to corresponding pixel values in permutation-based CIM's using a two-tailed t-test, FDR-corrected for multiple comparisons ($q < .10$). Informative CIM's were determined based on the presence of significant pixels in at least one CIEL*a*b* colour channel (see analysed CIM's in Fig. 1d). This procedure aims to help visualising CIM features relevant for face space topography and, more importantly, to select only features that can contribute meaningfully to image reconstruction.

Image reconstruction procedure. Perception-based reconstructions of unfamiliar faces were generated as follows. First, one unfamiliar face image was systematically left out, and a new face space construct was estimated based on the confusability matrix of the remaining 56 faces. Then, CIM's were derived for each dimension and significant features/dimensions were determined using the procedure above. Next, the overall face space construct, generated with the aid of all 57 unfamiliar faces, was aligned to the one above using Procrustes analysis and the resulting mapping function allowed the left-out target face to be projected in this space. Last, the target reconstruction was generated by summing an average face, obtained from 56 faces, with significant features weighted by the corresponding coordinates of the projected target (Fig. 1e). Thus, the reconstruction procedure capitalises on the ability to extract and select a limited number of informative CIM's, as described above.

Memory-based reconstructions of learned and famous faces were conducted in a similar manner with the difference that all 57 facial identities used in the perceptual task were used for the purpose of feature derivation (instead of 56 at a time) and memory targets were projected, one by one, in the space derived from all 57 faces. Also, similarity ratings in the memory-based

task were normalised such that their variance matched that of the ratings from the perception-based task separately for each participant.

Examples of reconstructed images for all categories of faces, unfamiliar, learned, or famous, are shown in Fig. 2. While corresponding stimuli for Fig2a, b could not be reproduced due to copyright restrictions, we note that stimuli were based, for Fig 2a, from top to bottom, on images labelled as *Rafd090_30_Caucasian_male_neutral_frontal* and *Rafd090_33_Caucasian_male_neutral_frontal* in the Radboud database[9], and *m-073-1* in the AR database[10]; for Fig 2b, they were based on images labelled as: *m-010-1* in the AR database[10], *Rafd090_07_Caucasian_male_neutral_frontal* in the Radboud database[9], and *88-11* in the FEI database[11].

References

1. Marks, D. F. New directions for mental imagery research. *J. Ment. Imag.* **19,** 153–167 (1995).
2. Duchaine, B. C. & Nakayama, K. The Cambridge Face Memory Test: Results for neurologically intact individuals and an investigation of its validity using inverted face stimuli and prosopagnosic participants. *Neuropsychologia* **44,** 576–585 (2006).
3. Watson, A. B. & Pelli, D. G. QUEST: A Bayesian adaptive psychometric method. *Percept. Psychophys.* **33,** 113–120 (1983).
4. Valentine, T. A unified account of the effects of distinctiveness, inversion, and race in face recognition. *Q. J. Exp. Psychol.* **43,** 161–204 (1991).
5. Nestor, A., Plaut, D. C. & Behrmann, M. Feature-based face representations and image reconstruction from behavioral and neural data. *Proc. Natl. Acad. Sci.* **113,** 416–421

(2016).

6. Murray, R. F. Classification images : A review. *J. Vis.* **11,** 1–25 (2011).

7. Smith, M. L., Gosselin, F. & Schyns, P. G. Measuring internal representations from behavioral and brain data. *Curr. Biol.* **22,** 191–196 (2012).

8. Neri, P. & Levi, D. M. Receptive versus perceptive fields from the reverse-correlation viewpoint. *Vision Res.* **46,** 2465–2474 (2006).

9. Langner, O. *et al.* Presentation and validation of the Radboud Faces Database. *Cogn. Emot.* **24,** 1377–1388 (2010).

10. Martinez, A. R. & Benavente, R. *The AR Face Database, CVC Technical Report #24*. (1998).

11. Thomaz, C. E. & Giraldi, G. A. A new ranking method for principal components analysis and its application to face image analysis. *Image Vis. Comput.* **28,** 902–913 (2010).
